# Integrative Analysis of the lncRNA-Associated ceRNA Regulatory Network Response to Hypoxia in Alveolar Type II Epithelial Cells of Tibetan Pigs

**DOI:** 10.3389/fvets.2022.834566

**Published:** 2022-02-08

**Authors:** Yanan Yang, Yongqing Li, Haonan Yuan, Xuanbo Liu, Yue Ren, Caixia Gao, Ting Jiao, Yuan Cai, Shengguo Zhao

**Affiliations:** ^1^College of Animal Science and Technology, Gansu Agricultural University, Lanzhou, China; ^2^Xinjiang Academy of Animal Sciences, Urumqi, China; ^3^Institute of Animal Husbandry and Veterinary Medicine, Academy of Agriculture and Animal Husbandry Sciences, Lhasa, China; ^4^State Key Laboratory of Veterinary Biotechnology, Harbin Veterinary Research Institute, Chinese Academy of Agricultural Sciences, Harbin, China; ^5^College of Grassland Science, Gansu Agricultural University, Lanzhou, China

**Keywords:** hypoxia, ATII cells, swine, ceRNA network, ssc-miR-20b/MSTRG.57127.1/ssc-miR-7-5p axis

## Abstract

The function of alveolar type II epithelial (ATII) cells is severely hampered by oxygen deficiency, and understanding the regulatory mechanisms controlling responses to hypoxia may assist in relieving injury induced by hypoxia. In this study, we cultured ATII cells from Tibetan pigs and Landrace pigs under hypoxic and normoxic environments to screen for differentially expressed (DE) lncRNAs, DEmiRNAs, and construct their associated ceRNA regulatory networks in response to hypoxia. Enrichment analysis revealed that target genes of DElncRNAs of Tibetan pigs and Landrace pig between the normoxic (TN, LN) and hypoxic (TL, LL) groups significantly enriched in the proteoglycans in cancer, renal cell carcinoma, and erbB signaling pathways, while the target genes of DEmiRNAs were significantly enriched in the axon guidance, focal adhesion, and mitogen-activated protein kinase (MAPK) signaling pathways. Hypoxia induction was shown to potentially promote apoptosis by activating the focal adhesion/PI3K-Akt/glycolysis pathway. The ssc-miR-20b/MSTRG.57127.1/ssc-miR-7-5p axis potentially played a vital role in alleviating hypoxic injury by regulating ATII cell autophagy under normoxic and hypoxic conditions. MSTRG.14861.4-miR-11971-z-CCDC12, the most affected axis, regulated numerous RNAs and may thus regulate ATII cell growth in Tibetan pigs under hypoxic conditions. The ACTA1/ssc-miR-30c-3p/MSTRG.23871.1 axis is key for limiting ATII cell injury and improving dysfunction and fibrosis mediated by oxidative stress in Landrace pigs. Our findings provide a deeper understanding of the lncRNA/miRNA/mRNA regulatory mechanisms of Tibetan pigs under hypoxic conditions.

## Introduction

The breeding of Tibetan pigs by humans can be traced back to the seventh century, and these animals adapted to hypoxic and low-pressure environments due to long-term natural selection on the Tibetan Plateau (1–3). Tibetan pigs harbor a distinct fungal community (4) and immunomodulatory function (5) and have developed organs that are adaptable to hypoxia induced by high altitudes to facilitate oxygen delivery under hypobaric hypoxic conditions. Alveolar type II (ATII) cells produce, secrete, and recycle a variety of pulmonary surfactant proteins and chemokines to recruit macrophages, eliminate inflammatory processes, and decrease surface tension as a protective barrier against possible infections and provide efficient ventilation (6). Alveolar type II cell apoptosis is induced by the alveolar lining layer; destructive exudative changes occur, and lung edema occurs in the early phase of hypoxia; conversely, reactive hyperplasia of ATII cells is predominant in the later phase of alveolar hypoxia (7). Increasing evidence suggests that alveolar epithelial cells undergo apoptosis during pathological hypoxic lung injury and fibrosis, which correlates with their reduced ability to proliferate and restore the alveolar architecture (8–10). The regulation of hypoxia-related genes enables cellular adaptation to a hypoxic environment via increased glucose metabolism (11), cell proliferation and migration (12), increased survival (13), as well as by the promotion of angiogenesis (14). Advances in sequencing technology have revealed mRNA expression levels are regulated by miRNAs, lncRNAs, or circRNAs, and analysis of these factors could provide comprehensive insight into the complicated cellular biological process of hypoxia as a rapid approach to reveal the regulatory network (15, 16). Existing literature shows that lncRNAs and miRNAs participate in diverse biological processes in mammals, such as development, growth, immunity, and reproduction (17–19). lncRNAs often bind to the proximal promoters of protein-coding genes to regulate protein expression, and lncRNA FAF overexpression was shown to significantly inhibit cardiomyocyte apoptosis induced by hypoxia (20). Moreover, lncRNA BCRT1 is upregulated in response to hypoxia and has potential as a biomarker and therapeutic target for breast cancer (21). RMRP regulates hypoxia-induced injury by modulating the p53 signaling pathway, which is a direct target of miR-214-5p (22). In Tibetan pigs, mounting research evidence has also shown that RNAs are abundantly expressed in the lung tissue transcriptome in response to hypoxia (23), but no integrated analyses of how their networks regulate ATII cells have been performed. Thus, we compared the expression levels of mRNAs, miRNAs, and lncRNAs in the ATII cells of Tibetan and Landrace pigs under normoxic and hypoxic conditions. Then, we constructed and preliminarily verified lncRNA/miRNA/mRNA regulatory networks to identify the key factors involved in the ATII cell response to hypoxia.

## Materials and Methods

### Sample Collection

Alveolar type II cell samples from Tibetan pigs (TN, *n* = 4) and Landrace pigs (LN, *n* = 4) were collected at 48 h after culture in 21% O_2_, 5% CO_2_, 79% N_2_, and 37°C under normoxic conditions, and control group samples from Tibetan pigs (TL, *n* = 4) and Landrace pigs (LL, *n* = 4) were acquired at parallel time points at 2% O_2_, 5% CO_2_, 98% N_2_, and 37°C under hypoxic conditions. Three of each group were flash-frozen in liquid nitrogen for RNA extraction, and the rest were used for the flow cytometric assay.

### Total RNA Isolation and Illumina Sequencing

Total RNA was isolated from ATII cells using TRIzol reagent kit (Invitrogen, Carlsbad, CA, USA), and RNA quality was assessed on an Agilent 2100 Bioanalyzer (Agilent Technologies, Palo Alto, CA, USA). All samples had an RNA integrity number (RIN) > 8. The enriched mRNAs and lncRNAs were reverse transcribed into cDNA, purified with the QiaQuick PCR extraction kit (Qiagen, Venlo, The Netherlands), end repaired, subjected to poly(A) addition, and ligated to Illumina sequencing adaptors. cDNA libraries were filtered and selected from the digested products and sequenced on the Illumina HiSeq^TM^ 4000 platform (or other platforms) by Gene Denovo Biotechnology Co. (Guangzhou, China).

RNA molecules were enriched for miRNA sequencing (miRNA-seq) by polyacrylamide gel electrophoresis (PAGE) at a size range of 18–30 nt. A 3′ adaptor was added to enrich the 36–44 nt RNAs, and the 5′ adaptor was then connected to the RNA. The ligation products were reverse transcribed by PCR amplification, and the 140–160 bp PCR products were enriched to generate a cDNA library and sequenced using Illumina HiSeq Xgten by Gene Denovo Biotechnology Co. (Guangzhou, China).

### Identification of DERNAs

The protocols for mRNA and lncRNA identification were as follows: reads were filtered by fastp (24) (version 0.18.0) to obtain clean reads and further mapped to the ribosomal RNA (rRNA) database by the short read alignment tool Bowtie 2 (25). The reads were removed from the rRNA and used to assemble and analyze the transcriptome. Paired-end clean reads were mapped to the *Sus scrofa* RefSeq using HISAT2 (26). Transcript reconstruction was carried out with StringTie software (version 1.3.4) (https://ccb.jhu.edu/software/stringtie/index.shtml) (27) and HISAT2 (http://ccb.jhu.edu/software/hisat/index.shtml) (28) to identify new genes and new splice variants of known genes. The protein-coding potential of novel transcripts was assessed using CNCI (29) and CPC (30), and their intersection was used to identify long non-coding RNAs. The expression abundances of mRNA and lncRNA were calculated according to fragment per kilobase of transcript per million mapped reads (FPKM) values using StringTie software. We used a false discovery rate (FDR) <0.05 and absolute fold change ≥2 as the thresholds for identifying differentially expressed mRNAs (DEmRNAs) and differentially expressed lncRNAs (DElncRNAs) using DESeq2 (31).

Potential miRNAs were identified from raw reads, filtered to generate clean reads and aligned with small RNAs in the GenBank database. Clean reads were aligned with small RNAs in the Rfam database (32) to identify and remove others, which were also aligned with the reference genome (*Sus scrofa*) and searched against the miRbase database (33) to identify known miRNAs. The miRNA expression levels were calculated and normalized to transcripts per million (TPM) values. We identified miRNAs with fold changes ≥2 and *P*-values <0.05 as significant DEmiRNAs.

### GO and KEGG Pathway Enrichment Analyses

The database for annotation, visualization, and integrated discovery (DAVID) was utilized to conduct Gene Ontology (GO) functional annotation (http://www.geneontology.org/) and Kyoto Encyclopedia of Genes and Genomes (KEGG) pathway (http://www.genome.jp/kegg/pathway.html) enrichment analyses to determine the roles, functions, and enrichment of different RNA biological pathways. GO terms and pathways with a *q*-value < 0.05 were considered significantly enriched.

### Integrated Analysis of lncRNAs, miRNAs, and mRNAs

The DElncRNAs, DEmiRNAs, and mRNAs were analyzed using DESeq2 and miRanda. Target genes of lncRNAs were identified by analyzing the correlation between the expression levels of lncRNAs and protein-coding genes by RNAplex. Target genes of miRNAs were predicted by RNAhybrid 89 (version 2.1.2) + svm_light (version 6.01), miRanda (version 3.3a), and TargetScan (version 7.0). Furthermore, a coexpression network diagram of the lncRNAs, miRNAs, and mRNAs was generated by assembling all coexpression competing triplets and visualized using Cytoscape software (34).

### Flow Cytometric Assay of Cell Apoptosis

Alveolar type II cells were collected and washed with PBS for the quantitative analysis of cell apoptosis. Furthermore, annexin V-FITC and 10 μl of PI staining solution were added to the cells, which were resuspended in Annexin V-FITC binding buffer at room temperature in the dark for 20 min. A BD FACSCanto II flow cytometer (BD Biosciences, San Jose, CA, USA) was used to analyze cell apoptosis, and the fractions of the cell populations in different quadrants were analyzed using quadrant statistics.

### Quantitative Real-Time-qPCR Analysis

To verify the RNA-seq results, first-strand cDNA was synthesized from RNA samples returned by transcriptome sequencing using a FastQuant cDNA first-strand synthesis kit (TianGen, China), and cDNA was used as the template for gene expression analysis. qPCR was performed on a LightCycler 96 Real-Time System (Roche, Switzerland) using SYBR® Premix Ex Taq™ II (TaKaRa, China). Eight DElncRNAs, four DEmiRNAs, and six DEmRNAs were selected to determine the reliability of the data, and the amplification primers are listed (Supplementary Table 1 of Supplementary Material 1).

## Results

### Overview of RNA Sequencing

**Figure 1:**
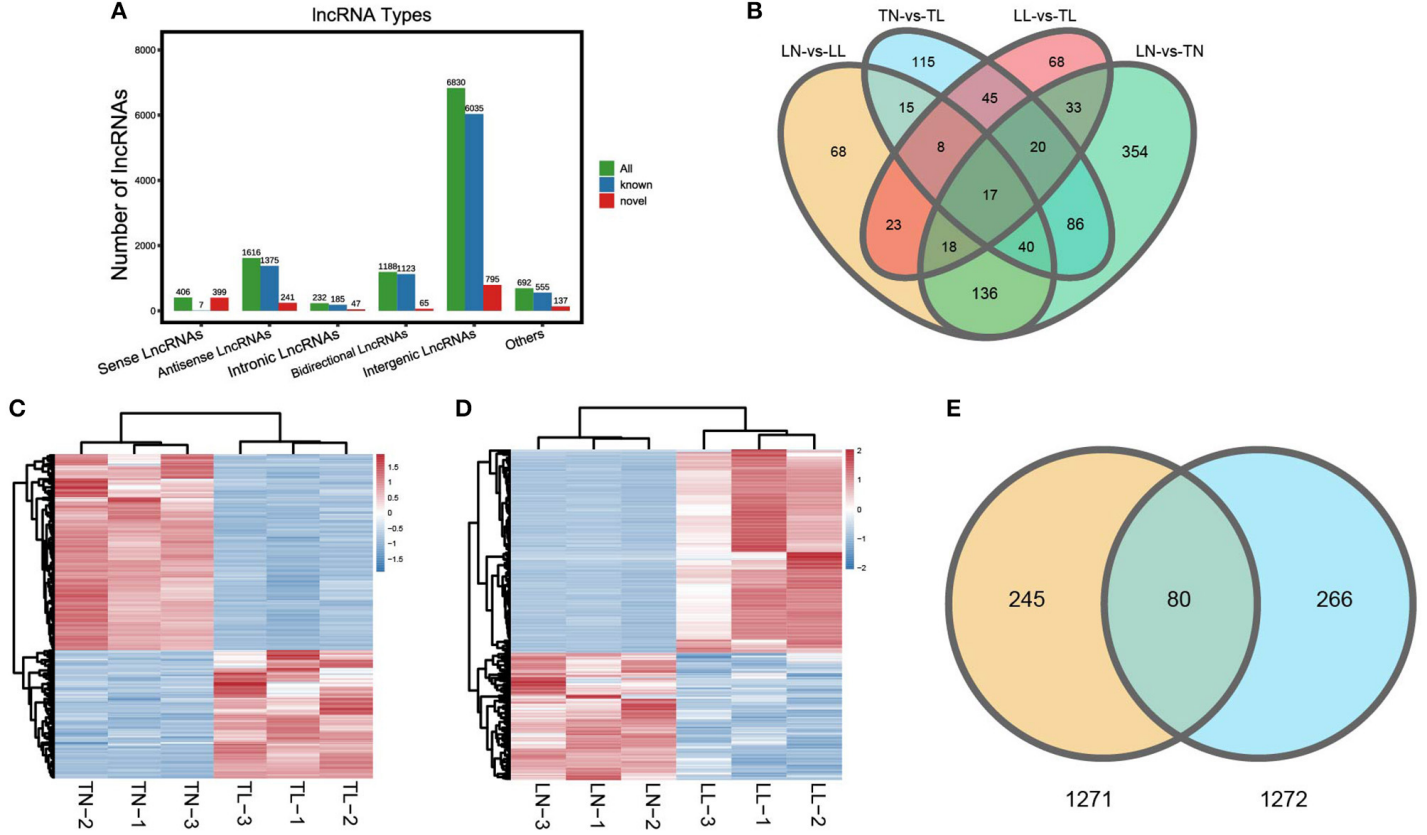
Categories and features of the lncRNAs in ATII cells of Tibetan pigs and Landrace pigs. **(A)** Categories and types of lncRNAs originating from different genomic sites. **(B)** Venn diagram of lncRNAs interactions based on the overlapping DElncRNAs. **(C)** Heatmap of lncRNAs in ATII cells between the TN and TL groups. **(D)** Heatmap of lncRNAs in ATII cells between the LN and LL groups. **(E)** Common DElncRNAs shared between the normoxic (TN and LN) and hypoxic (TL and LL) groups.

Twelve cDNA libraries were sequenced for lncRNA-seq and mRNA-seq analysis of ATII cells (Supplementary Figure 1 of Supplementary Material 2). Averages of 100,489,128, 97,339,785, 64,417,965, and 44,846,728 raw reads were acquired from the TN, TL, LN, and LL groups, respectively. The raw reads, clean reads, clean bases, error rates, Q20 values, Q30 values, and GC contents of each library are shown (Supplementary Table 2 of Supplementary Material 1). After quality filtering, an average of 44,821,226 clean reads were mapped to the porcine reference genome. lncRNAs were classified based on their genomic location, and the 10,964 lncRNAs (9,280 known lncRNAs and 1,684 novel lncRNAs) consisted of 62.30% long intergenic non-coding RNAs and 14.74% antisense lncRNAs but a minimal of intronic lncRNAs (Figure 1A; Supplementary Table 3 of Supplementary Material 1). Another 12 libraries were constructed for miRNA-seq analysis of ATII cells. A total of 9,947,404–13,467,206 raw reads were generated, and 9,847,843–13,350,909 high-quality RNA sequences were obtained after removing reads at a suitable level, which accounted for more than 98.86% of the clean reads (Supplementary Table 4 of Supplementary Material 1).

**Figure 2:**
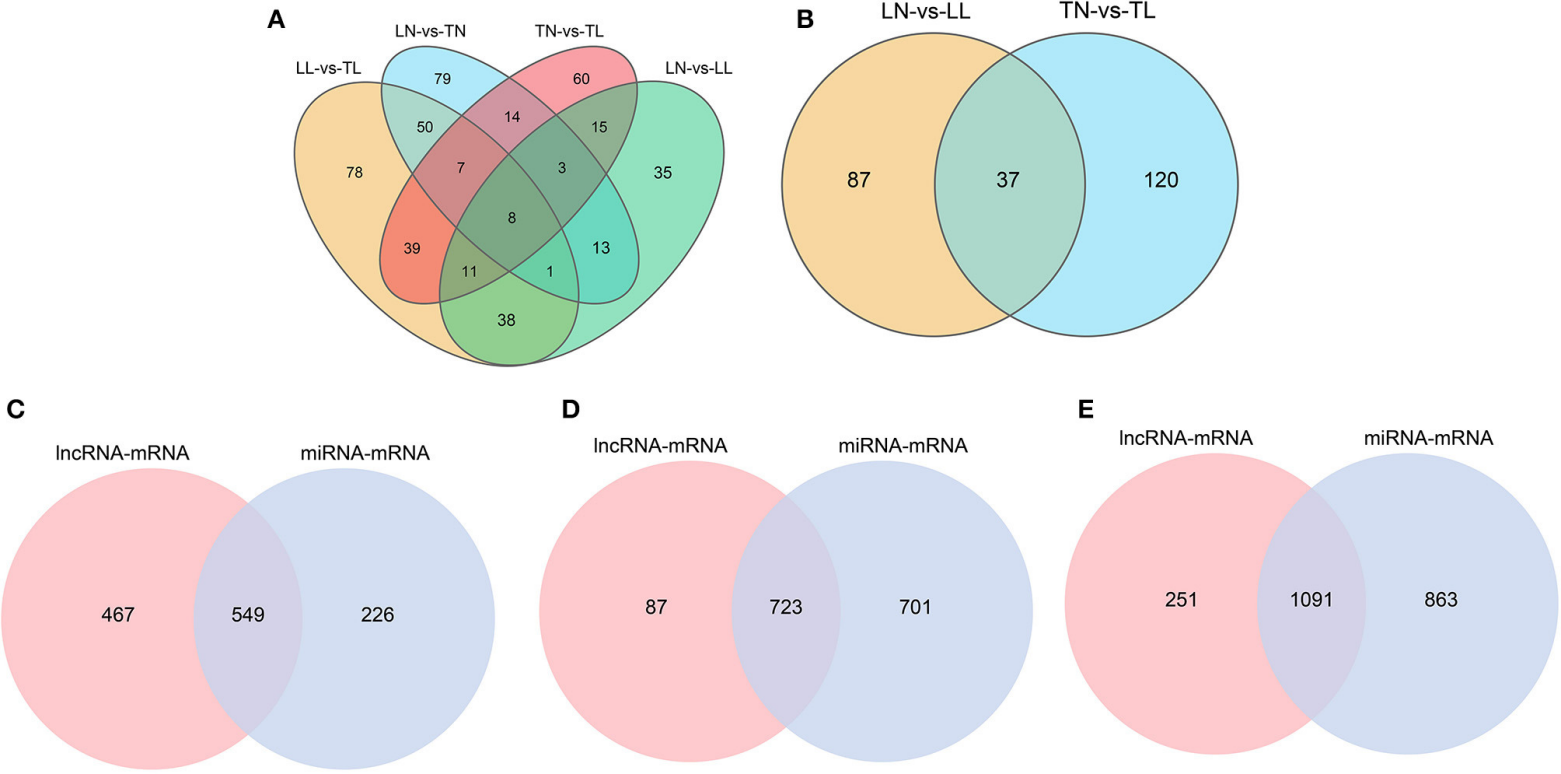
Features of the miRNAs in ATII cells among the four groups. **(A)** Venn diagram of miRNAs interactions based on the overlapping DEmiRNAs. **(B)** Common DEmiRNAs shared between the normoxic (TN and LN) and hypoxic (TL and LL) groups. **(C)** Venn diagram of mRNAs interactions from lncRNA-mRNA and miRNA-mRNA pairs between the normoxic (TN and LN) and hypoxic (TL and LL) groups. **(D)** Venn diagram of mRNAs interactions from lncRNA-mRNA and miRNA-mRNA pairs between the TN and TL groups. **(E)** Venn diagram of mRNAs interactions from lncRNA-mRNA and miRNA-mRNA pairs between the LN and LL groups.

A total of 325 DElncRNAs (200 up- and 125 downregulated) and 124 DEmiRNAs (78 up- and 46 downregulated) were identified in the LN group compared to the LL group (Figures 1B, 2A). Cluster analysis of DElncRNAs was conducted, and the results are shown as a heatmap (Figures 1C,D). Then, the most interesting candidates, including 80 DElncRNAs and 37 DEmiRNAs were identified and screened based on the intersection between the normoxic (TN vs. TL) and hypoxic (TL vs. LL) groups to assess the regulation of RNA responses to hypoxia (Figures 1E, 2B). Eight lncRNAs, four miRNAs, and six mRNAs were randomly selected and detected using quantitative real-time (qRT)-PCR to validate the results (Supplementary Figure 2 of Supplementary Material 2).

### Prediction of DElncRNA- and DEmiRNA-Targeted mRNAs

In total, 1,716 (1,616 lncRNA and 1,054 mRNA) and 72 (43 lncRNA and 29 mRNA) lncRNA-mRNA pairs from total lncRNAs and DElncRNAs were obtained among the four groups (Supplementary Materials 3). The 2,679 target mRNAs of 157 DEmiRNAs were analyzed between the TN and TL groups (Supplementary Materials 4). Specifically, the target mRNAs of DElncRNAs and DEmiRNAs between the normoxic (TN vs. TL) and hypoxic (TL vs. LL) groups were examined to further investigate the potential lncRNA–mRNA and miRNA–mRNA interactions in response to hypoxia induction and to establish the potential roles of the lncRNAs and miRNAs in hypoxia adaptation (Figure 2C).

### Functional Annotation of Target mRNAs

**Figure 3:**
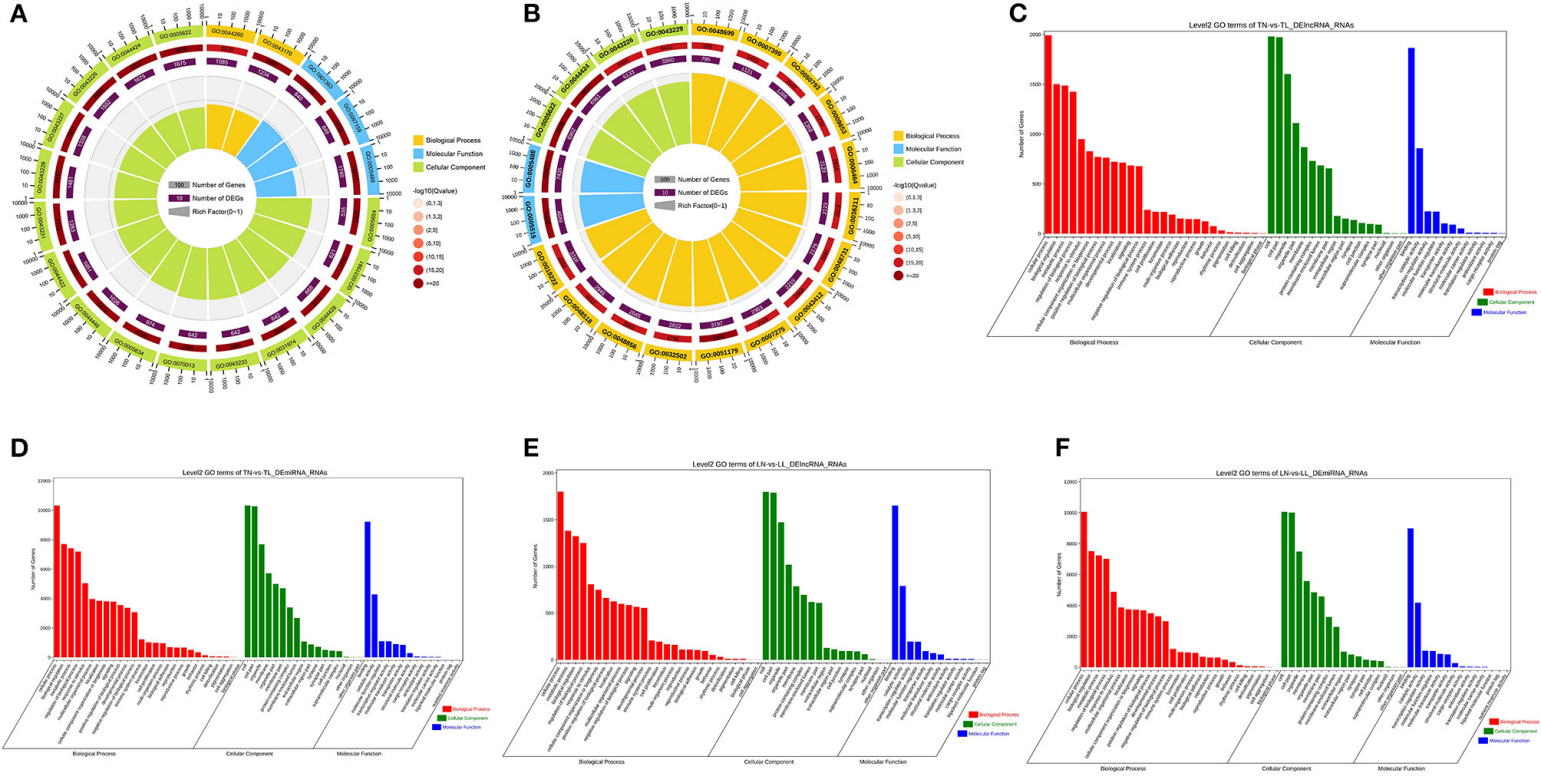
GO functional annotation. Common target mRNAs of DElncRNAs **(A)** and DEmiRNAs **(B)** between the normoxic (TN and LN) and hypoxic (TL and LL) groups. Target mRNAs of DElncRNAs **(C)** and DEmiRNAs **(D)** between the TN and TL groups. Target mRNAs of DElncRNAs **(E)** and DEmiRNA **(F)** of Landrace pigs between the LN and LL groups.

To further investigate the potential functions of DEmiRNAs, we performed GO enrichment and KEGG pathway analyses of their target mRNAs among the four groups (Supplementary Figure 3 of Supplementary Material 2). In this study, numerous target mRNAs of DElncRNAs (DEmiRNAs) were significantly enriched for the intracellular part (intracellular), binding (binding), and macromolecule metabolic process (localization) of cellular component, molecular function, and biological process between the normoxic (TN, LN) and hypoxic (TL, LL) groups, respectively (Figures 3A,B). The target mRNAs of DElncRNAs were enriched in 62 and 61 GO categories; moreover, the target mRNAs of DEmiRNAs were enriched in 135 and 130 categories between the Tibetan pigs (excluding DEmiRNAs shared between the normoxia and hypoxia groups) and Landrace pigs (excluding DEmiRNAs shared between the normoxia and hypoxia groups) at different oxygen concentrations (Figures 3C–F).

**Figure 4:**
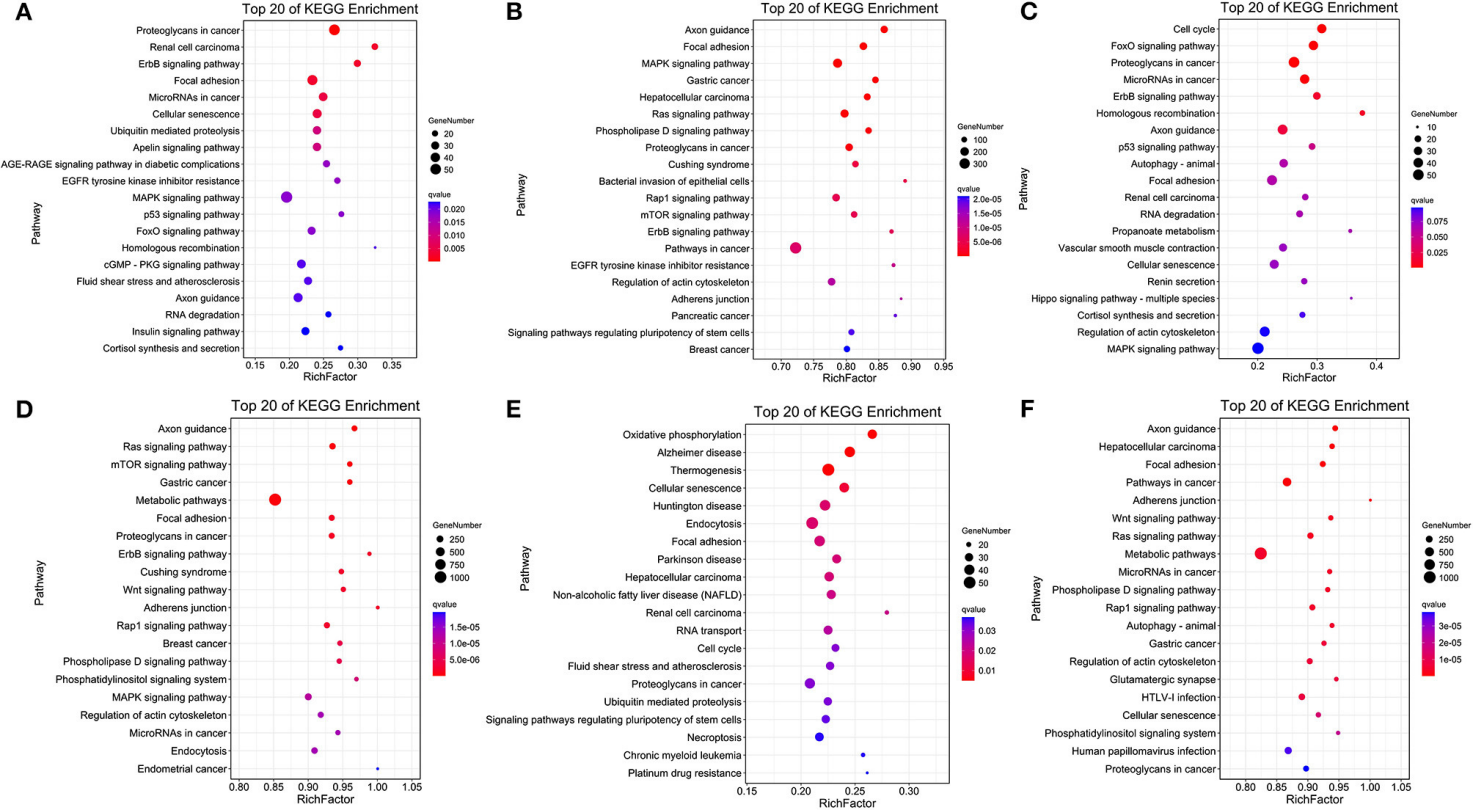
KEGG pathway enrichment analysis. Common target mRNAs of DElncRNAs **(A)** and DEmiRNAs **(B)** between the normoxic (TN and LN) and hypoxic (TL and LL) groups. Target mRNAs of DElncRNAs **(C)** and DEmiRNAs **(D)** between the TN and TL groups. Target mRNAs of DElncRNAs **(E)** and DEmiRNA **(F)** between the LN and LL groups.

The comparison of normoxic (TN, LN) and hypoxic (TL, LL) groups revealed that the target genes of DElncRNAs were significantly enriched in the proteoglycans in cancer, renal cell carcinoma, and erbB signaling pathways, while the target genes of DEmiRNAs were significantly enriched in the axon guidance, focal adhesion, and MAPK signaling pathways (Figures 4A,B). Interestingly, the cell cycle, FOXO signaling, and proteoglycans in cancer pathways were significantly enriched among the target mRNAs of the DElncRNAs; however, the axon guidance, ras signaling, and mTOR signaling pathways were significantly enriched among the target mRNAs of the DEmiRNAs between the TN and TL groups (excluding DEmiRNAs shared between the normoxia and hypoxia groups) (Figures 4C,D). Numerous target mRNAs of the DElncRNAs were significantly enriched in the oxidative phosphorylation, Alzheimer's disease, and thermogenesis pathways; the target mRNAs of the DEmiRNAs were significantly enriched in the axon guidance, hepatocellular carcinoma, and focal adhesion pathways between the LN and LL groups (excluding DEmiRNAs shared between the normoxia and hypoxia groups) (Figures 4E,F).

### lncRNA-miRNA-mRNA Networks Profiles

**Figure 5:**
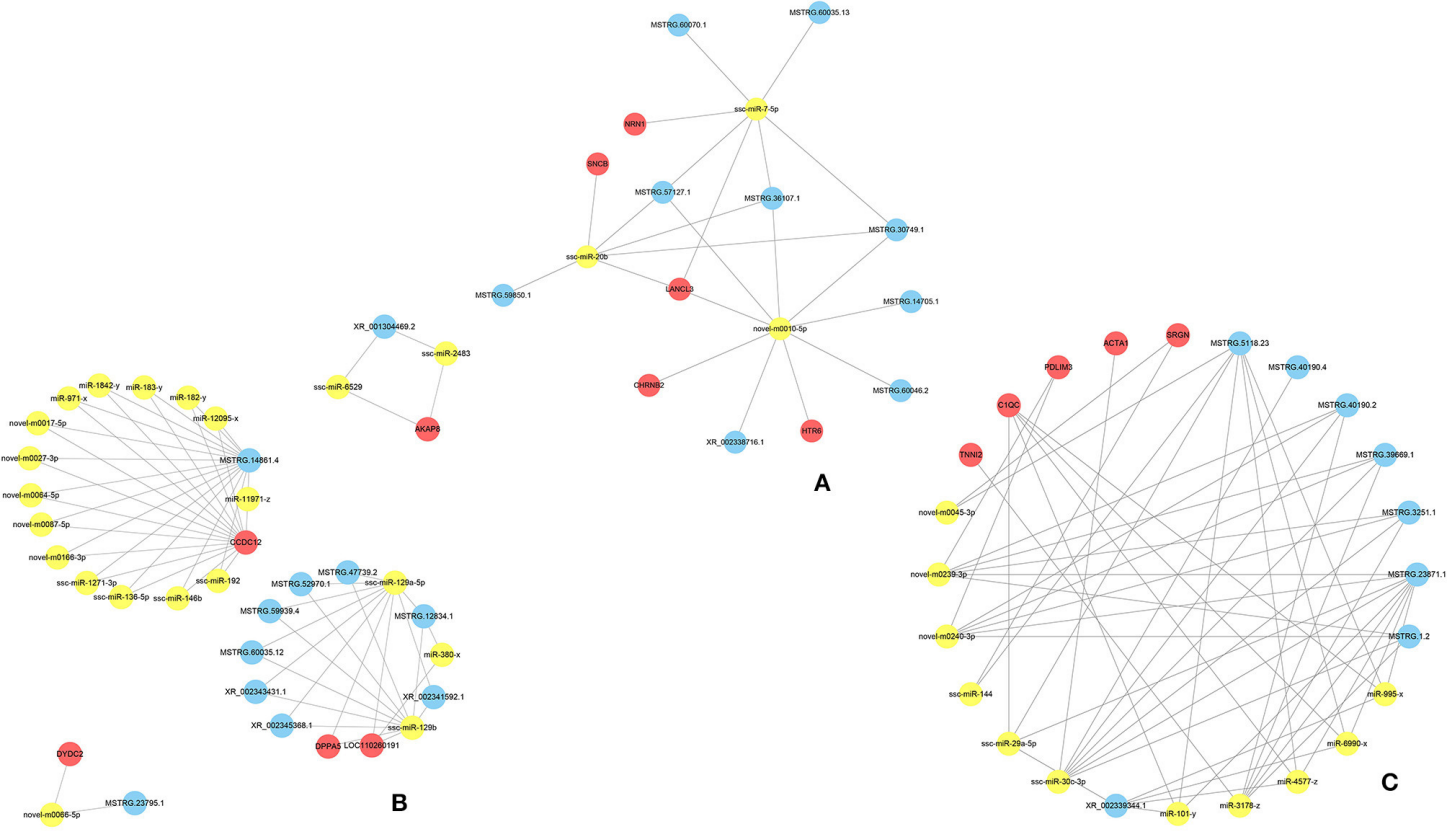
The ceRNA coregulation network. The predicted lncRNA-miRNA-mRNA network constructed based on the lncRNA-mRNA and miRNA-mRNA pairs **(A)** between the normoxic (TN and LN) and hypoxic (TL and LL) groups, **(B)** TN and TL groups, and **(C)** LN and LL groups.

Three-component (lncRNA/miRNA/mRNA) ceRNA regulatory networks were constructed to identify the most effected RNAs associated with hypoxic adaptation in the ATII cells of Tibetan pigs using intersecting mRNAs of lncRNA–mRNA pairs and miRNA–mRNA pairs (Figures 2C–E). Three networks were constructed between (1) the normoxic (TN, LN) and hypoxic (TL, LL) groups, (2) the TN and TL groups (excluding DEmRNAs shared between the normoxia and hypoxia groups), and (3) the LN and LL groups (excluding DEmRNAs shared between the normoxia and hypoxia groups), and the top five gene pairs in each network were revealed. The network between normoxic (TN, LN) and hypoxic (TL, LL) groups contained fifteen lncRNA–miRNA pairs and seven miRNA–mRNA pairs, including nine lncRNAs, three miRNAs, and five mRNAs (Supplementary Materials 5). The network between the TN and TL groups (excluding DEmRNAs shared between the normoxia and hypoxia groups) indicate an association among 11 lncRNA nodes. Twenty-three miRNA nodes, five mRNA nodes, and 58 edges, including ssc-miR-129b, CCDC12, and ssc-miR-129a-5p, were selected as the most affected RNAs. Here, the network of Landrace pigs (LN vs. LL) was composed of 38 lncRNA-miRNA pairs and 11 mRNA-miRNA pairs, 8 lncRNA nodes, 11 miRNAs node, and 5 mRNAs node, including ssc-miR-30c-3p, C1QC, and MSTRG.23871.1, which were selected as the most affected RNAs (Figure 5).

### Apoptosis of ATII Cells

**Figure 6:**
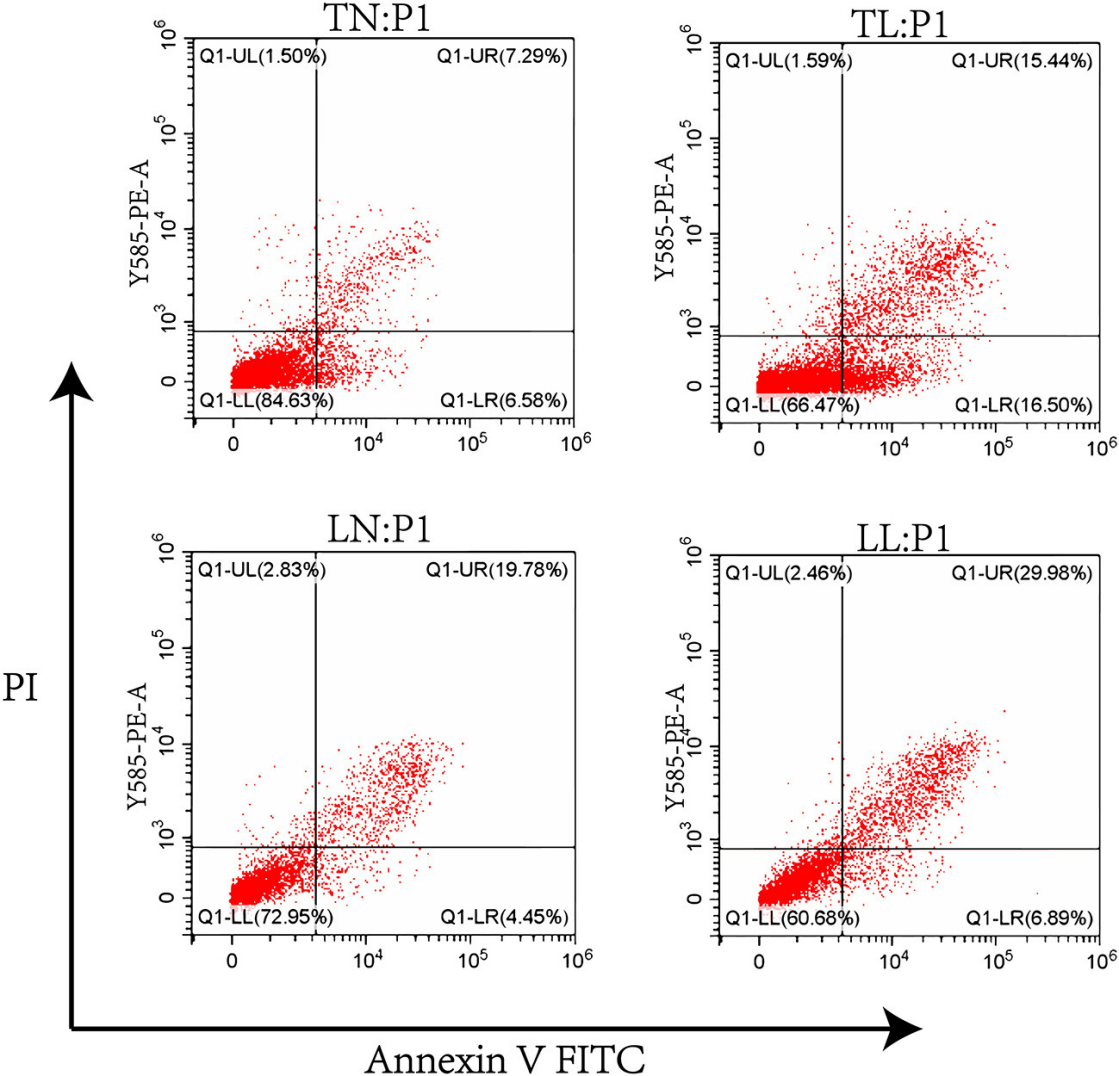
The apoptosis of ATII cells from Tibetan pigs and Landrace pigs under normoxic (21% O_2_) and hypoxic (2% O_2_) condition was determined.

Cell apoptosis was investigated by flow cytometric assays, and our results showed that cell apoptosis was higher in the hypoxic groups (TL, LL) than in the normoxic groups (TN, LN). Notably, the rate of cell apoptosis in Tibetan pigs was lower than that in Landrace pigs under the same conditions. The order of total apoptosis rates among the four groups was as follows: TN < LN < TL < LL (Figure 6).

## Discussion

Under hypoxic conditions, the development of domestic mammals, including Tibetan pigs (35), yaks (36), and Tibetan sheep (3), is severely hampered. The hearts (37), lungs (38), and deciduous teeth (39) of Tibetan pigs adapted better than those of any other pig; these pigs were originally found exclusively on the Tibetan Plateau, which has an average altitude of 4,268 m above sea level (40). A total of 1,716 lncRNA–mRNA pairs were obtained to analyze their associated key regulatory pathways among the four groups (TL, LL, TN, LN), and the result could help to elucidate the regulatory mechanisms of ATII cells in Tibetan pigs in response to hypoxia and provide a theoretical basis for formulating strategies to relieve disease and injury during hypoxia (41). Simultaneously, it is important to understand the responses of ATII cells to hypoxia to reveal a series of adaptation activities in the lung. Hypoxia induces pulmonary injury via cell apoptosis, which leads to transdifferentiation of ATII cells; hence, the regeneration of ATII cells is an important indicator of hypoxia damage (42). Here, we generated comprehensive ceRNA network profiles, including the lncRNAs, miRNAs, and mRNAs of ATII cells. The comparisons between Tibetan pigs and Landrace pigs under normoxic and hypoxic conditions revealed genes and pathways that possibly undergo adaptive changes in response to hypoxia. Notably, the ATII cells of Tibetan pigs and Landrace pigs showed some genes with the same expression patterns and some with different expression patterns, and the majority of DERNAs were involved in network regulation.

### Hypoxia Induction May Promote Apoptosis by Activating of the Focal Adhesion/PI3K-Akt/Glycolysis Pathway

**Figure 7:**
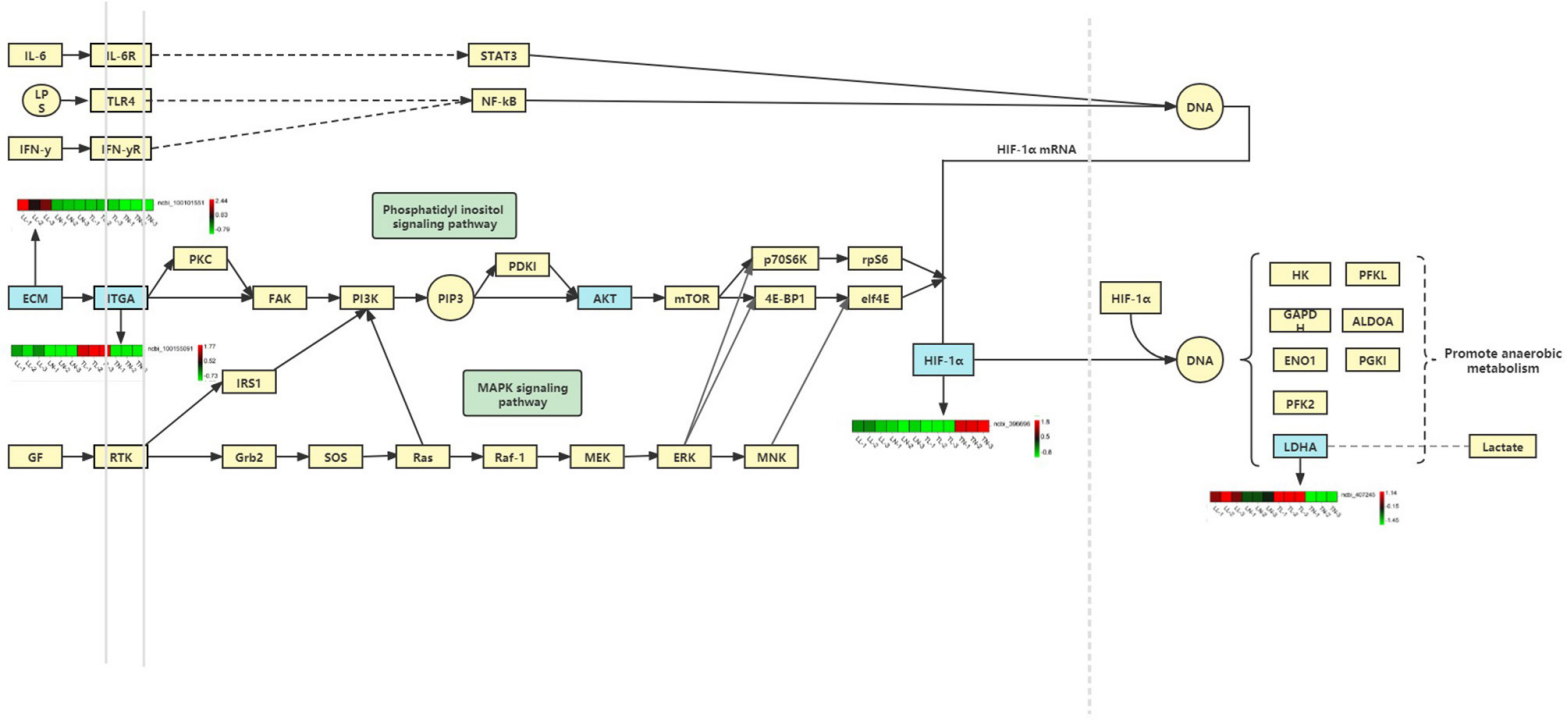
Hypoxia induction may promote apoptosis by activating of the focal adhesion/PI3K-Akt/glycolysis pathway.

Notably, the erbB signaling pathway, focal adhesion, and cellular senescence were clearly associated with different oxygen concentrations in ATII cells and enriched for a number of target mRNAs of DElncRNAs between the normoxic and hypoxic groups (Figure 7). Collagens that interact with integrins are widely represented in focal adhesion pathways, and focal adhesion kinases (FAKs) are focal adhesion complexes that play a key role in cell-substrate adhesions (43). Various inflammatory mediators and their receptors significant upregulate and affects normal life activities via damage the cell function caused by low oxygen levels (44, 45). In our analysis, we found *COL2A1* to be the target gene of ssc-miR-7-5p, miR-9277-z, and miR-148-z; the upregulation of this gene, along with *COL6A3, COL1A2* (target genes of miR-409-y, ssc-miR-1285, and miR-11980-z), and *COL5A1* (target gene of ssc-miR-139-3p, miR-1307-x, and miR-1197-y), indicated the enrichment of the focal adhesion pathway; these genes may link the extracellular matrix (ECM) and cytoskeleton in cells under hypoxia (46). Moreover, α and β chains form integrins that mediate differential cell interactions with specific ECM and cellular surface components, which are linked to arginine-glycine-aspartate (RGD) amino acid motifs as heterodimeric transmembrane adhesion receptors (47). Focal adhesion kinases are activated by integrins and integrate integrin signals by directly binding to signaling molecules, and FAK phosphorylation is important for the maintenance of normal cell adhesion (43, 48). We also identified *ITGA5* (target gene of miR-28-x, miR-411-y, ssc-miR-1285), *ITGA6*, and *ITGAL* (target gene of ssc-miR-326, ssc-miR-1, ssc-miR-9860-5p) as being significantly differentially expressed (DE) between the normoxic and hypoxic groups, and these are downstream genes related to the ECM, proteoglycans in cancer and focal adhesion pathways. *ITGA5* expression was significantly higher in the TL group than in the TN group, but was not significantly different in the LN and LL groups, indicating that it may influence and regulate the apoptosis, differentiation, migration, and proliferation of ATII cells in Tibetan pigs (49). In our study, the differential expression of protein kinase B among the four groups may have been mediated by ITGA5, which is also enriched in the PI3K-Akt signaling pathway and regulates a wide range of cellular activities, including cell proliferation (50), apoptosis (51), and metabolic progression (52). The antisense lncRNA MSTRG.36013.14 was complementary to the target gene AKT3, and its level was significantly higher in the TN and LL groups than that in the TL and LN groups; this lncRNA may regulate the phosphorylation of some vital downstream targets as the central mediator of the PI3K/Akt signaling pathway. Furthermore, the PI3K/Akt/mTOR (53) and VEGF/PI3K/Akt (54) signaling pathways regulate the expression of HIF-1α, which is closely associated with the concentration of oxygen in the environment and regulated by the PI3K/Akt signaling pathways as a downstream protein (55). The antioxidant function of enzymes could be activated following adaptive regulation to remove accumulated ROS in animals exposed to hypoxia as the main defense system (56). LDHA (the target genes of ssc-miR-429, ssc-miR-141, and miR-101-y) was expressed at a higher level in the hypoxia (TL and LL) groups than in the normoxia (TN and LN) groups and was significantly different in the TN and TL groups; this gene could be regulated by HIF-1α, and increased LDH expression may have promoted the accumulation of lactic acid in anaerobic glycolysis under the condition of hypoxia and further increased apoptosis, leading to numerous lung diseases in the hypoxic groups (TL and LL) (43, 57, 58). In our enrichment analysis, we found that exposure to hypoxia was mainly associated with the intracellular component GO term, and P4HA1, which could be induced by *HIF*-1α or hypoxia-independent factors, is a part of a gene expression signature associated with hypoxia and glycolysis in ATII cells (59, 60). *P*4*HA*1 and *P*4*HA*2 were the prolyl hydroxylase subtypes with significantly increased expression in cells under hypoxic conditions (61–63), which is similar to the results of our study, indicating that P4HA1 mediates hypoxia-induced invasion and migration (64).

### lncRNA-miRNA-mRNA Networks in ATII Cells Under Hypoxic Conditions

In general, lncRNAs and miRNAs can maintain mRNA stability and regulate gene silencing and transcription. Cellular hypoxia responses involve the activation of complex signaling pathways, many of which lead to transcriptional cascades that are designed to alter cellular transcriptomes and proteomes to optimally combat hypoxia-induced damage. Many genes specifically increase related transcriptional activities and regulate a series of metabolic activities in the body during long-term adaptation to hypoxic environments. MiR-7-5p is a fascinating miRNA that plays diverse roles in tumor suppression; it inhibits the invasion abilities of tumor cells by directly targeting *PI3K*/*Akt, FAK*, and *KLF*4 expression and may be a useful therapeutic target for the diagnosis and treatment of patients with glioblastoma (65–67). miR-20b inhibits the expressions of autophagy-related proteins that are induced by hypoxia/reoxygenation injury via ULK1 (68). The central genes ssc-miR-7-5p, ssc-miR-20b, and novel-m0010-5p intersected at MSTRG.57127.1, MSTRG.36107.1, and LANCL3 between the hypoxia (TL and LL) and normoxia (TN and LN) groups, which may indicate that the ssc-miR-20b/MSTRG.57127.1/ssc-miR-7-5p axis plays a vital role in alleviating hypoxia-related injury by inhibiting the proliferation of tumor cells or regulating ATII cell autophagy (66). MSTRG.14861.4-miR-11971-z-CCDC12, the most affected axis, regulated numerous RNAs between the TN and TL groups, and thus may regulate ATII cell growth under hypoxic conditions (69). MSTRG.23871.1, miR-3178-z, and ssc-miR-30c-3p were identified as the most influential genes and regulated multiple RNAs. The ACTA1/ssc-miR-30c-3p/MSTRG.23871.1 axis is key for limiting ATII cell injury and improving dysfunction and fibrosis mediated by oxidative stress in Landrace pigs (70). The regulation of the expression levels of these factors by ceRNAs may improve the adaptation of Tibetan pigs vs. Landrace pigs to hypoxic environments.

## Conclusion

In the present study, the ceRNA network of lncRNAs, miRNAs, and mRNAs from the ATII cells of Tibetan pigs and Landrace pigs under hypoxic environments was analyzed using whole-transcriptome techniques. Different regulatory ceRNA networks indicated that the MSTRG.14861.4/miR-11971-z/CCDC12 axis efficiently promotes ATII cell growth in Tibetan pigs under hypoxic conditions. In addition, oxidative stress may induce cell injury, increased dysfunction, and fibrosis in the ATII cells of Landrace pigs, but this could be relieved by the ACTA1/ssc-miR-30c-3p/MSTRG.23871.1 axis. These results may explain why Tibetan pigs adapted better to the plateau environment than Landrace pigs and will help to prevent hypoxia-induced injury in other mammals.

## Data Availability Statement

The datasets presented in this study can be found in online repositories. The names of the repository/repositories and accession number(s) can be found in the article/Supplementary Material.

## Ethics Statement

The animal study was reviewed and approved by 2006-398.

## Author Contributions

SZ was the overall project leader who provided financial support and experimental conception. YY was involved in data analyses, statistical analyses, language revisions, journal selection, and manuscript submissions and revisions. YL and HY contributed to the experimental design and implementation. CG contributed to the supervision and assistance of students in managing animals and collecting and analyzing samples. XL and YR were responsible for the trial implementation, supervision of students collecting and analyzing samples, and manuscript preparation. YC and TJ contributed to supervision of sample collection and analysis and manuscript editing. All authors contributed to the article and approved the submitted version.

## Funding

This study was supported by the National Natural Science Foundation of China (32060730, 31760644).

## Conflict of Interest

The authors declare that the research was conducted in the absence of any commercial or financial relationships that could be construed as a potential conflict of interest.

## Publisher's Note

All claims expressed in this article are solely those of the authors and do not necessarily represent those of their affiliated organizations, or those of the publisher, the editors and the reviewers. Any product that may be evaluated in this article, or claim that may be made by its manufacturer, is not guaranteed or endorsed by the publisher.

## Abbreviations

DElncRNAs: differentially expressed lncRNAs
DEmiRNAs: differentially expressed miRNAs
DEmRNAs: differentially expressed mRNAs
TN: ATII cells of Tibetan pigs were cultured under 21% O_2_
TL: ATII cells of Tibetan pigs were cultured under 2% O_2_
LN: ATII cells of Landrace pigs were cultured under 21% O_2_
LL: ATII cells of Landrace pigs were cultured under 2% O_2_.

## References

[1] Ma YF Han XM Huang CP Zhong L Adeola AC Irwin DM. Population genomics analysis revealed origin and high-altitude adaptation of Tibetan pigs. Sci Rep. (2019) 9:11463. 10.1038/s41598-019-47711-6 31391504 PMC6685962

[2] Ai H Fang X Yang B Huang Z Chen H Mao L. Adaptation and possible ancient interspecies introgression in pigs identified by whole-genome sequencing. Nat Genet. (2015) 47:217–25. 10.1038/ng.3199 25621459

[3] Wu DD Yang CP Wang MS Dong KZ Yan DW Hao ZQ. Convergent genomic signatures of high-altitude adaptation among domestic mammals. Natl Sci Rev. (2020) 7:952–63. 10.1093/nsr/nwz213 34692117 PMC8288980

[4] Li J Chen D Yu B He J Huang Z Mao X. The fungal community and its interaction with the concentration of short-chain fatty acids in the faeces of Chenghua, Yorkshire and Tibetan pigs. Microb Biotechnol. (2020) 13:509–21. 10.1111/1751-7915.13507 31691493 PMC7017814

[5] Cramer CL Patterson A Alchakaki A Soubani AO. Immunomodulatory indications of azithromycin in respiratory disease: a concise review for the clinician. Postgrad Med. (2017) 129:493–9. 10.1080/00325481.2017.1285677 28116959

[6] Guillamat-Prats R Puig F Camprubí-Rimblas M Herrero R Serrano-Mollar A GóMEZ MN. Intratracheal instillation of alveolar type II cells enhances recovery from acute lung injury in rats. J Heart Lung Transplant. (2018) 37:782–91. 10.1016/j.healun.2017.10.025 29229270

[7] Sulkowska M. Morphological studies of the lungs in chronic hypobaric hypoxia. Pol J Pathol. (1997) 48:225–34. 9529928

[8] Bernard O Jeny F Uzunhan Y Dondi E Terfous R Label R. Mesenchymal stem cells reduce hypoxia-induced apoptosis in alveolar epithelial cells by modulating HIF and ROS hypoxic signaling. Am J Physiol Lung Cell Mol Physiol. (2018) 314:L360–71. 10.1152/ajplung.00153.2017 29167125

[9] Guan R Wang J Li D Li Z Liu H Ding M. Hydrogen sulfide inhibits cigarette smoke-induced inflammation and injury in alveolar epithelial cells by suppressing PHD2/HIF-1α/MAPK signaling pathway. Int Immunopharmacol. (2020) 81:105979. 10.1016/j.intimp.2019.105979 31771816

[10] Sherman MA Suresh MV Dolgachev VA McCandless LK Xue X Ziru L. Molecular characterization of hypoxic alveolar epithelial cells after lung contusion indicates an important role for HIF-1α. Ann Surg. (2018) 267:382–91. 10.1097/SLA.0000000000002070 27811509 PMC6010036

[11] Huang M Yang L Peng X Wei S Fan Q Yang S. Autonomous glucose metabolic reprogramming of tumour cells under hypoxia: opportunities for targeted therapy. J Exp Clin Cancer Res. (2020) 39:185. 10.1186/s13046-020-01698-5 32928258 PMC7491117

[12] You B Liu Y Chen J Huang X Peng H Liu Z. Vascular peroxidase 1 mediates hypoxia-induced pulmonary artery smooth muscle cell proliferation, apoptosis resistance and migration. Cardiovasc Res. (2018) 114:188–99. 10.1093/cvr/cvx234 29186367

[13] Criscuoli M Ulivieri C Filippi I Monaci S Guerrini G Crifò B. The Shc protein Rai enhances T-cell survival under hypoxia. J Cell Physiol. (2020) 235:8058–70. 10.1002/jcp.29461 31944299

[14] Dulloo I Hooi PB Sabapathy K. Hypoxia-induced DNp73 stabilization regulates Vegf-a expression and tumor angiogenesis similar to TAp73. Cell Cycle. (2015) 14:3533–9. 10.1080/15384101.2015.1078038 26267146 PMC4825702

[15] Zhao X Tang DY Zuo X Zhang TD Wang C. Identification of lncRNA-miRNA-mRNA regulatory network associated with epithelial ovarian cancer cisplatin-resistant. J Cell Physiol. (2019) 234:19886–94. 10.1002/jcp.28587 30950060

[16] Li W Ma S Bai X Pan W Ai L Tan W. Long noncoding RNA WDFY3-AS2 suppresses tumor progression by acting as a competing endogenous RNA of microRNA-18a in ovarian cancer. J Cell Physiol. (2020) 235:1141–54. 10.1002/jcp.29028 31347170

[17] Lei GL Niu Y Cheng SJ Li YY Bai ZF Yu LX. Upregulation of long noncoding RNA W42 promotes tumor development by binding with DBN1 in hepatocellular carcinoma. World J Gastroenterol. (2021) 27:2586–602. 10.3748/wjg.v27.i20.2586 34092977 PMC8160624

[18] Wang Y. Wang P. Zhang Y. Xu J. Li Z. Li Z.. (2020). Decreased expression of the host long-noncoding RNA-GM facilitates viral escape by inhibiting the kinase activity TBK1 via S-glutathionylation. Immunity. 53, 1168–1181.e7. 10.1016/j.immuni.2020.11.010 33326766

[19] Reza A Choi YJ Han SG Song H Park C Hong K. Roles of microRNAs in mammalian reproduction: from the commitment of germ cells to peri-implantation embryos. Biol Rev Camb Philos Soc. (2019) 94:415–38. 10.1111/brv.12459 30151880 PMC7379200

[20] Shi HJ Wang MW Sun JT Wang H Li YF Chen BR. A novel long noncoding RNA FAF inhibits apoptosis via upregulating FGF9 through PI3K/AKT signaling pathway in ischemia-hypoxia cardiomyocytes. J Cell Physiol. (2019) 234:21973–87. 10.1002/jcp.28760 31093967

[21] Liang Y Song X Li Y Chen B Zhao W Wang L. LncRNA BCRT1 promotes breast cancer progression by targeting miR-1303/PTBP3 axis. Mol Cancer. (2020) 19:85. 10.1186/s12943-020-01206-5 32384893 PMC7206728

[22] Teng Y Ding M Wang X Li H Guo Q Yan J. LncRNA RMRP accelerates hypoxia-induced injury by targeting miR-214-5p in H9c2 cells. J Pharmacol Sci. (2020) 142:69–78. 10.1016/j.jphs.2019.07.014 31839421

[23] Yang Y Yuan H Yang T Li Y Gao C Jiao T. The expression regulatory network in the lung tissue of Tibetan pigs provides insight into hypoxia-sensitive pathways in high-altitude hypoxia. Front Genet. (2021) 12:691592. 10.3389/fgene.2021.691592 34691141 PMC8529057

[24] Chen S Zhou Y Chen Y Gu J. Fastp: an ultra-fast all-in-one FASTQ preprocessor. Bioinformatics (Oxford, England). (2018) 34:i884–90. 10.1093/bioinformatics/bty560 30423086 PMC6129281

[25] Langmead B Salzberg SL. Fast gapped-read alignment with Bowtie 2. Nat Methods. (2012) 9:357–9. 10.1038/nmeth.1923 22388286 PMC3322381

[26] Kim D Langmead B Salzberg SL. HISAT: a fast spliced aligner with low memory requirements. Nat Methods. (2015) 12:357–12, 360. 10.1038/nmeth.3317 25751142 PMC4655817

[27] Pertea M Pertea GM Antonescu CM Chang TC Mendell JT Salzberg SL. StringTie enables improved reconstruction of a transcriptome from RNA-seq reads. Nat Biotechnol. (2015) 33:290–5. 10.1038/nbt.3122 25690850 PMC4643835

[28] Pertea M Kim D Pertea GM Leek JT Salzberg SL. Transcript-level expression analysis of RNA-seq experiments with HISAT, StringTie and Ballgown. Nat Protoc. (2016) 11:1650–67. 10.1038/nprot.2016.095 27560171 PMC5032908

[29] Sun L Luo H Bu D Zhao G Yu K Zhang C. Utilizing sequence intrinsic composition to classify protein-coding and long non-coding transcripts. Nucleic Acids Res. (2013) 41:e166. 10.1093/nar/gkt646 23892401 PMC3783192

[30] Kong L Zhang Y Ye ZQ Liu XQ Zhao SQ Wei L. CPC: assess the protein-coding potential of transcripts using sequence features and support vector machine. Nucleic Acids Res. (2007) 35:W345–9. 10.1093/nar/gkm391 17631615 PMC1933232

[31] Li B Dewey CN. RSEM: accurate transcript quantification from RNA-Seq data with or without a reference genome. BMC Bioinformatics. (2011) 12:323. 10.1186/1471-2105-12-323 21816040 PMC3163565

[32] Griffiths-Jones S Bateman A Marshall M Khanna A Eddy SR. Rfam: an RNA family database. Nucleic Acids Res. (2003) 31:439–41. 10.1093/nar/gkg006 12520045 PMC165453

[33] Griffiths-Jones S Grocock RJ Van DS Bateman A Enright AJ. MiRBase: microRNA sequences, targets and gene nomenclature. Nucleic Acids Res. (2006) 34:D140–4. 10.1093/nar/gkj112 16381832 PMC1347474

[34] Szklarczyk D Franceschini A Wyder S Forslund K Heller D Huerta-Cepas J. STRING v10: protein-protein interaction networks, integrated over the tree of life. Nucleic Acids Res. (2015) 43:D447–52. 10.1093/nar/gku1003 25352553 PMC4383874

[35] Xin J Zhang H He Y Duren Z Bai C Chen L. Chromatin accessibility landscape and regulatory network of high-altitude hypoxia adaptation. Nat Commun. (2020) 11:4928. 10.1038/s41467-020-18638-8 33004791 PMC7529806

[36] Ge Q Guo Y Zheng W Cai Y Qi X Zhao S. A comparative analysis of differentially expressed mRNAs, miRNAs and circRNAs provides insights into the key genes involved in the high-altitude adaptation of yaks. BMC Genomics. (2021) 22:744. 10.1186/s12864-021-08044-9 34654374 PMC8518315

[37] Yang Y Gao C Yang T Sha Y Cai Y Wang X. Vascular characteristics and expression of hypoxia genes in Tibetan pigs' hearts. Vet Med Sci. (2022) 8:177–86. 10.1002/vms3.639 34561963 PMC8788992

[38] Yang Y Gao C Yang T Sha Y Cai Y Wang X. Characteristics of Tibetan pig lung tissue in response to a hypoxic environment on the Qinghai-Tibet Plateau. Arch Anim Breed. (2021) 64:283–92. 10.5194/aab-64-283-2021 34235247 PMC8253108

[39] Lu T Zheng Y Yang H Wu B Xiong J Huang C. Structural characteristics of the deciduous teeth of Tibetan miniature pigs. Nan Fang Yi Ke Da Xue Xue Bao. (2019) 39:1113–7. 10.12122/j.issn.1673-4254.2019.09.18 31640964 PMC8888271

[40] Li M Tian S Jin L Zhou G Li Y Zhang Y. Genomic analyses identify distinct patterns of selection in domesticated pigs and Tibetan wild boars. Nat Genet. (2013) 45:1431–8. 10.1038/ng.2811 24162736

[41] Fan JL Zhu TT Xue ZY Ren WQ Guo JQ Zhao HY. LncRNA-XIST protects the hypoxia-induced cardiomyocyte injury through regulating the miR-125b-hexokianse 2 axis. In Vitro Cell Dev Biol Anim. (2020) 56:349–57. 10.1007/s11626-020-00459-0 32415544

[42] McClendon J Jansing NL Redente EF Gandjeva A Ito Y Colgan SP. Hypoxia-inducible factor 1α signaling promotes repair of the alveolar epithelium after acute lung injury. Am J Pathol. (2017) 187:1772–86. 10.1016/j.ajpath.2017.04.012 28618253 PMC5530913

[43] Lu Q Rounds S. Focal adhesion kinase and endothelial cell apoptosis. Microvasc Res. (2012) 83:56–63. 10.1016/j.mvr.2011.05.003 21624380 PMC3189508

[44] Lin N Shay JES Xie H Lee DSM Skuli N Tang Q. Myeloid cell hypoxia-inducible factors promote resolution of inflammation in experimental colitis. Front Immunol. (2018) 9:2565. 10.3389/fimmu.2018.02565 30455703 PMC6230677

[45] Saad A Zhu XY Herrmann S Hickson L Tang H Dietz AB. Adipose-derived mesenchymal stem cells from patients with atherosclerotic renovascular disease have increased DNA damage and reduced angiogenesis that can be modified by hypoxia. Stem Cell Res Ther. (2016) 7:128. 10.1186/s13287-016-0389-x 27612459 PMC5016873

[46] Choi HJ Sanders TA Tormos KV Ameri K Tsai JD Park AM. ECM-dependent HIF induction directs trophoblast stem cell fate via LIMK1-mediated cytoskeletal rearrangement. PLoS ONE. (2013) 8:e56949. 10.1371/journal.pone.0056949 23437279 PMC3578927

[47] Campbell ID Humperies MJ. Integrin structure, activation, and interactions. Cold Spring Harb Perspect Biol. (2011) 3:a004994. 10.1101/cshperspect.a004994 21421922 PMC3039929

[48] Zhao X Guan JL. Focal adhesion kinase and its signaling pathways in cell migration and angiogenesis. Adv Drug Deliv Rev. (2011) 63:610–5. 10.1016/j.addr.2010.11.001 21118706 PMC3132829

[49] Cui S Yang CL Chen DY. LncRNA EWSAT1 regulates the tumorigenesis of NSCLC as a ceRNA by modulating miR-330-5p/ITGA5 axis. Biochem Genet. (2021) 59:1441–56. 10.1007/s10528-021-10069-4 33928467

[50] Deng Y Wan Q Yan W. Integrin α5/ITGA5 promotes the proliferation, migration, invasion and progression of oral squamous carcinoma by epithelial-mesenchymal transition. Cancer Manag Res. (2019) 11:9609–20. 10.2147/CMAR.S223201 32009816 PMC6859091

[51] Yu M Chu S Fei B Fang X Liu Z. O-GlcNAcylation of ITGA5 facilitates the occurrence and development of colorectal cancer. Exp Cell Res. (2019) 382:111464. 10.1016/j.yexcr.2019.06.009 31202709

[52] Li L Qu Y Mao M Xiong Y Mu D. The involvement of phosphoinositid 3-kinase/Akt pathway in the activation of hypoxia-inducible factor-1alpha in the developing rat brain after hypoxia-ischemia. Brain Res. (2008) 1197:152–8. 10.1016/j.brainres.2007.12.059 18241842

[53] Yang X Gao M Miao M Jiang C Zhang D Yin Z. Combining combretastatin A4 phosphate with ginsenoside Rd synergistically inhibited hepatocellular carcinoma by reducing HIF-1α via PI3K/AKT/mTOR signalling pathway. J Pharm Pharmacol. (2021) 73:263–71. 10.1093/jpp/rgaa006 33793802 PMC8531789

[54] Wang D Zhao W Liu J Wang Y Yuan C Zhang F. Effects of HIF-1α on spermatogenesis of varicocele rats by regulating VEGF/PI3K/Akt signaling pathway. Reprod Sci. (2021) 28:1161–74. 10.1007/s43032-020-00395-0 33237516

[55] Gao T Zhang X Zhao J Zhou F Wang Y Zhao Z. SIK2 promotes reprogramming of glucose metabolism through PI3K/AKT/HIF-1α pathway and Drp1-mediated mitochondrial fission in ovarian cancer. Cancer Lett. (2020) 469:89–101. 10.1016/j.canlet.2019.10.029 31639424

[56] Ghosh S Kamble NU Verma P Salvi P Petla BP Roy S. Arabidopsis protein l-ISOASPARTYL METHYLTRANSFERASE repairs isoaspartyl damage to antioxidant enzymes and increases heat and oxidative stress tolerance. J Biol Chem. (2020) 295:783–99. 10.1074/jbc.RA119.010779 31831624 PMC6970934

[57] Zhao LL Wu H Sun JL Liao L Cui C Liu Q. MicroRNA-124 regulates lactate transportation in the muscle of largemouth bass (micropterus salmoides) under hypoxia by targeting MCT1. Aquat Toxicol. (2020) 218:105359. 10.1016/j.aquatox.2019.105359 31765944

[58] Hu D Linders A Yamak A Correia C Kijlstra JD Garakani A. Metabolic maturation of human pluripotent stem cell-derived cardiomyocytes by inhibition of HIF1α and LDHA. Circ Res. (2018) 123:1066–79. 10.1161/CIRCRESAHA.118.313249 30355156 PMC6208155

[59] Gilkes DM Bajpai S Chaturvedi P Wirtz D Semenza GL. Hypoxia-inducible factor 1 (HIF-1) promotes extracellular matrix remodeling under hypoxic conditions by inducing P4HA1, P4HA2, and PLOD2 expression in fibroblasts. J Biol Chem. (2013) 288:10819–29. 10.1074/jbc.M112.442939 23423382 PMC3624462

[60] Chen L Shen YH Wang X Wang J Gan Y Chen N. Human prolyl-4-hydroxylase alpha (I) transcription is mediated by upstream stimulatory factors. J Biol Chem. (2006) 281:10849–55. 10.1074/jbc.M511237200 Epub 2006 Feb 17 16488890 PMC2819823

[61] Morimoto C Takedachi M Kawasaki K Shimomura J Murata M Hirai A. Hypoxia stimulates collagen hydroxylation in gingival fibroblasts and periodontal ligament cells. J Periodontol. (2021) 92:1635–45. 10.1002/JPER.20-0670 33660864

[62] Trackman PC. Diverse biological functions of extracellular collagen processing enzymes. J Cell Biochem. (2005) 96:927–37. 10.1002/jcb.20605 16167328 PMC1352157

[63] Hofbauer KH Gess B Lohaus C Meyer HE Katschinski D Kurt Z. Oxygen tension regulates the expression of a group of procollagen hydroxylases. Eur J Biochem. (2003) 270:4515–22. 10.1046/j.1432-1033.2003.03846.x 14622280

[64] Zhu X Liu S Yang X Wang W Shao W Ji T. P4HA1 as an unfavorable prognostic marker promotes cell migration and invasion of glioblastoma via inducing EMT process under hypoxia microenvironment. Am J Cancer Res. (2021) 11:590–617. 33575089 PMC7868758

[65] Yin CY Kong W Jiang J Xu H Zhao W. miR-7-5p inhibits cell migration and invasion in glioblastoma through targeting SATB1. Oncol Lett. (2019) 17:1819–25. 10.3892/ol.2018.9777 30675243 PMC6341908

[66] Xiao H. MiR-7-5p suppresses tumor metastasis of non-small cell lung cancer by targeting NOVA2. Cell Mol Biol Lett. (2019) 24:60. 10.1186/s11658-019-0188-3 31832068 PMC6864997

[67] Tian S Chen M Wang B Han Y Shang H Chen J. MiR-7-5p promotes hepatic stellate cell activation by targeting fibroblast growth factor receptor 4. Gastroenterol Res Pract. (2020) 2020:5346573. 10.1155/2020/5346573 32587612 PMC7303738

[68] Lu Y Wang S Cai S Gu X Wang J Yang Y. Propofol-induced miR-20b expression initiates endogenous cellular signal changes mitigating hypoxia/re-oxygenation-induced endothelial autophagy *in vitro*. Cell Death Dis. (2020) 11:681. 10.1038/s41419-020-02828-9 32826852 PMC7442825

[69] Fan C Dong L Zhu N Xiong Y Zhang J Wang L. Isolation of siRNA target by biotinylated siRNA reveals that human CCDC12 promotes early erythroid differentiation. Leuk Res. (2012) 36:779–83. 10.1016/j.leukres.2011.12.017 22269669

[70] Ning BB Zhang Y Wu DD Cui JG Liu L Wang PW. Luteolin-7-diglucuronide attenuates isoproterenol-induced myocardial injury and fibrosis in mice. Acta Pharmacol Sin. (2017) 38:331–41. 10.1038/aps.2016.142 28112175 PMC5342667

