# Supplementary material for: Integrative Analysis of the lncRNA-Associated ceRNA Regulatory Network Response to Hypoxia in Alveolar Type II Epithelial Cells of Tibetan Pigs
Source: Front Vet Sci. 2022 Feb 8;9:834566. doi: 10.3389/fvets.2022.834566 (PMC8861501; doi:10.3389/fvets.2022.834566)
Supplement: Supplementary file 1 [file Data_Sheet_1.doc]

Table S1 Primers used to detect DEGs in ATII cells of pigs by qRT-PCR

| **Genes** | **Primer sequences (5'-3')** |
| --- | --- |
| *B3GALT1* | F: ATGTCAAGCAAGAAACATCTCC |
|  | R: TTTACAAAACCCCTTCACCA |
| *HK2* | F: GATGGGACAGAACACGGAG |
|  | R: CATGAAGTTAGCCAGGCACT |
| *G2E3* | F: GTCAAGTGGAATTTGGCAGAG |
|  | R: TCGTTTACATCGGGGTGC |
| *DDX60* | F: AGAAGAACCCATCACCAATCA |
|  | R: ACTCGTTAGGAAAGGCAAATC |
| *POLR3G* | F: CAATGGCTGGGAATAAAGG |
|  | R: CAGGAAATAGTGGAGGTGGTT |
| *SASS6* | F: AATGTGGTTGAGGGTAGACTGA |
|  | R: TTTTGGCAGCACAAGGGT |
| *β-actin* | F: CAGTCGGTTGGATGGAGCAT |
|  | R: AGGCAGGGACTTCCTGTAAC |
| *SHANK3* | F: GCCCGTGTCCTGCTTTT |
|  | R: TCCGAATCTTTGTGGGTCTT |
| miR-1-y | CCGGCGTGGAATGTAAAGAAGTATGT |
| ssc-miR-218 | CCGCTTGTGCTTGATCTAACCATGT |
| novel-m0008-5p | TATAGGAGAAGCCGGCGGG |
| novel-m0009-5p | CAACCCGGTCAGCCTCC |
| *U6* | F: GGAACGATACAGAGAAGATTAGC |
|  | R: TGGAACGCTTCACGAATTTGCG |
| MSTRG.2648.4 | F: CTTAGCAAGCCCCAACAGG |
|  | R: TGATGTGGTCAGGCATTTAGTT |
| MSTRG.34022.6 | F: AGCACTTGCCCATTTGTCC |
|  | R: GGTCCTATCCTACTGTCATCCC |
| MSTRG.26453.14 | F: CGCTTGAAAACAGGCACC |
|  | R: CCCAAAACCAATCGAGGAT |
| MSTRG.34025.1 | F: TTACTTCCTGAAACCAAAACACT |
|  | R: CCCTGCTCCAATTACTGCT |
| MSTRG.37989.1 | F: GCCAATTCTGGAGAACAACC |
|  | R: AACCACAGGGCTAAGTAGGG |
| MSTRG.37990.1 | F: AGTCCAGGAGCGGTCAAG |
|  | R: CCACCCAGCACCCAAAG |
| MSTRG.56367.13 | F: GAAAATAAAGTAAGAGCCACGAA |
|  | R: CAGATAAGGGAGCCATAGCAT |
| MSTRG.59589.2 | F: GAAAATAAAGTAAGAGCCACGAA |
|  | R: CAGATAAGGGAGCCATAGCAT |

Table S2 Overview of the reads and quality filtering of mRNA and lncRNA libraries

| Sample | RawDatas | CleanData(%) | AF_Q20 | AF_Q30 | AF_GC |
| --- | --- | --- | --- | --- | --- |
| LL-1 | 62403492 | 60866934 (97.54%) | 9173313324 | 8811997105 | 4236573072 |
| LL-2 | 25633118 | 24508662 (95.61%) | 3777680765 | 3645029380 | 1735618420 |
| LL-3 | 46503574 | 44840052 (96.42%) | 6834630271 | 6581418310 | 3208746606 |
| LN-1 | 61799310 | 59986428 (97.07%) | 9070593725 | 8712775720 | 4372610179 |
| LN-2 | 63897660 | 62162356 (97.28%) | 9362285143 | 8974106859 | 4551749810 |
| LN-3 | 67556926 | 65719906 (97.28%) | 9913641106 | 9516406381 | 4758709867 |
| TL-1 | 102037632 | 99724036 (97.73%) | 14973512308 | 14379653290 | 7259467329 |
| TL-2 | 98882120 | 96772832 (97.87%) | 14520839322 | 13949795079 | 6974155924 |
| TL-3 | 91099604 | 89034416 (97.73%) | 13371144377 | 12820447271 | 6411590130 |
| TN-1 | 117092830 | 115082170 (98.28%) | 17151172274 | 16457397257 | 8313005466 |
| TN-2 | 89798010 | 88022768 (98.02%) | 13142387754 | 12587436052 | 6336147586 |
| TN-3 | 94576544 | 92895658 (98.22%) | 13880053597 | 13357991075 | 6687507426 |

Table S3 lncRNA types

| Types | All | known | novel |
| --- | --- | --- | --- |
| Sense lncRNAs | 406 | 7 | 399 |
| Antisense lncRNAs | 1616 | 1375 | 241 |
| Intronic lncRNAs | 232 | 185 | 47 |
| Bidirectional lncRNAs | 1188 | 1123 | 65 |
| Intergenic lncRNAs | 6830 | 6035 | 795 |
| Others | 692 | 555 | 137 |

Table S4 Overview of the reads and quality filtering of miRNA libraries

| Sample | Clean_reads | High_quality | Clean_tags | Total_abundance | Exist_mirna_abundance |
| --- | --- | --- | --- | --- | --- |
| TL-1 | 13467206 | 13350909 | 11341418 | 11341418 | 7111780 (62.71%) |
| TL-2 | 12806586 | 12699876 | 10781288 | 10781288 | 6626211 (61.46%) |
| TL-3 | 11995365 | 11894344 | 9735117 | 9735117 | 6144917 (63.12%) |
| TN-1 | 11915084 | 11817122 | 9430821 | 9430821 | 6677737 (70.81%) |
| TN-2 | 11761674 | 11587554 | 8191420 | 8191420 | 5545509 (67.70%) |
| TN-3 | 9947404 | 9847843 | 6403578 | 6403578 | 4362294 (68.12%) |
| LL-1 | 10234648 | 10117911 | 6707800 | 6707800 | 2502087 (37.30%) |
| LL-2 | 11285907 | 11211379 | 8984049 | 8984049 | 5483229 (61.03%) |
| LL-3 | 13175380 | 13049934 | 10404999 | 10404999 | 6038382 (58.03%) |
| LN-1 | 11031384 | 10913186 | 8641550 | 8641550 | 4097970 (47.42%) |
| LN-2 | 14071448 | 13925252 | 10772275 | 10772275 | 5349321 (49.66%) |
| LN-3 | 10892875 | 10781661 | 8503588 | 8503588 | 4222866 (49.66%) |
