# Supplementary material for: Integrative Analysis of the lncRNA-Associated ceRNA Regulatory Network Response to Hypoxia in Alveolar Type II Epithelial Cells of Tibetan Pigs
Source: Front Vet Sci. 2022 Feb 8;9:834566. doi: 10.3389/fvets.2022.834566 (PMC8861501; doi:10.3389/fvets.2022.834566)
Supplement: Supplementary file 2 [file Data_Sheet_2.doc]

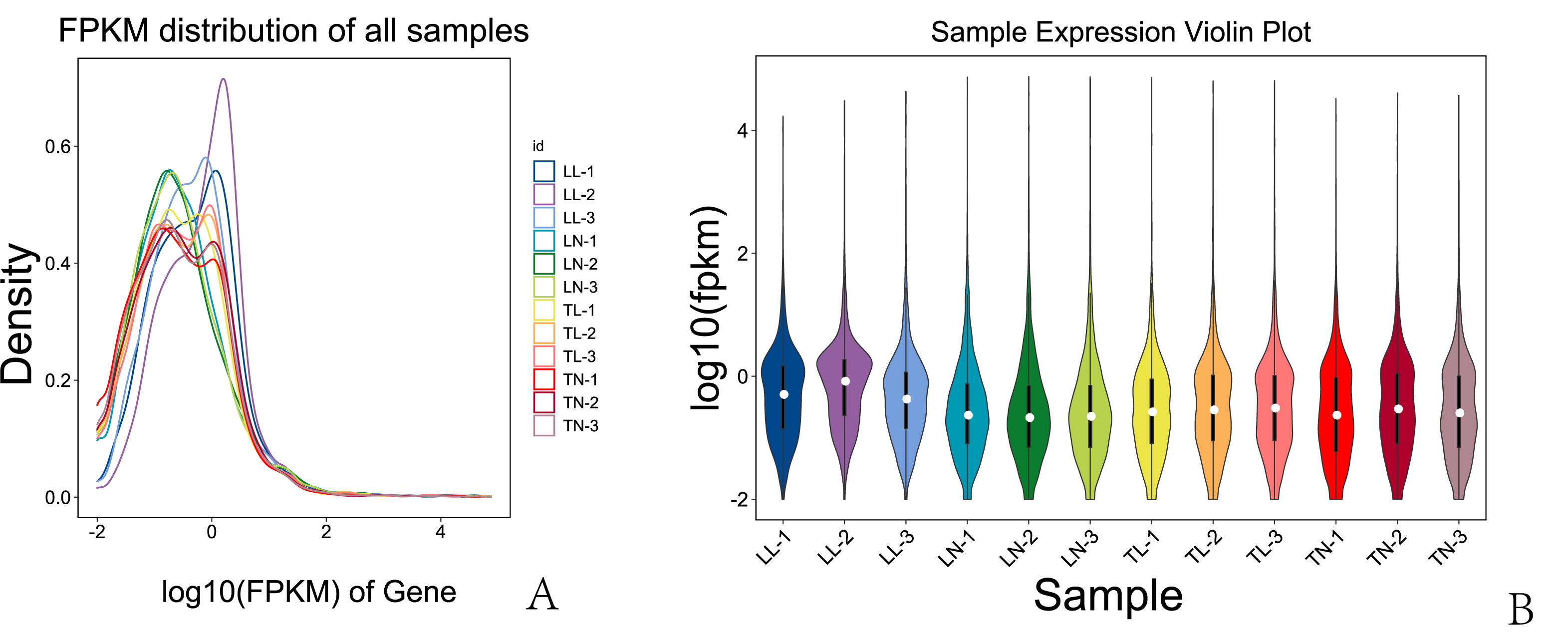


**Figure S1** A. Comparison of the overall expression levels among four groups. B. Differential expression of lncRNAs among four groups. P-values and log2FC values were used to screen for differentially expressed transcripts according to the following thresholds: *P* < 0.05 and |log2FC| > 1.


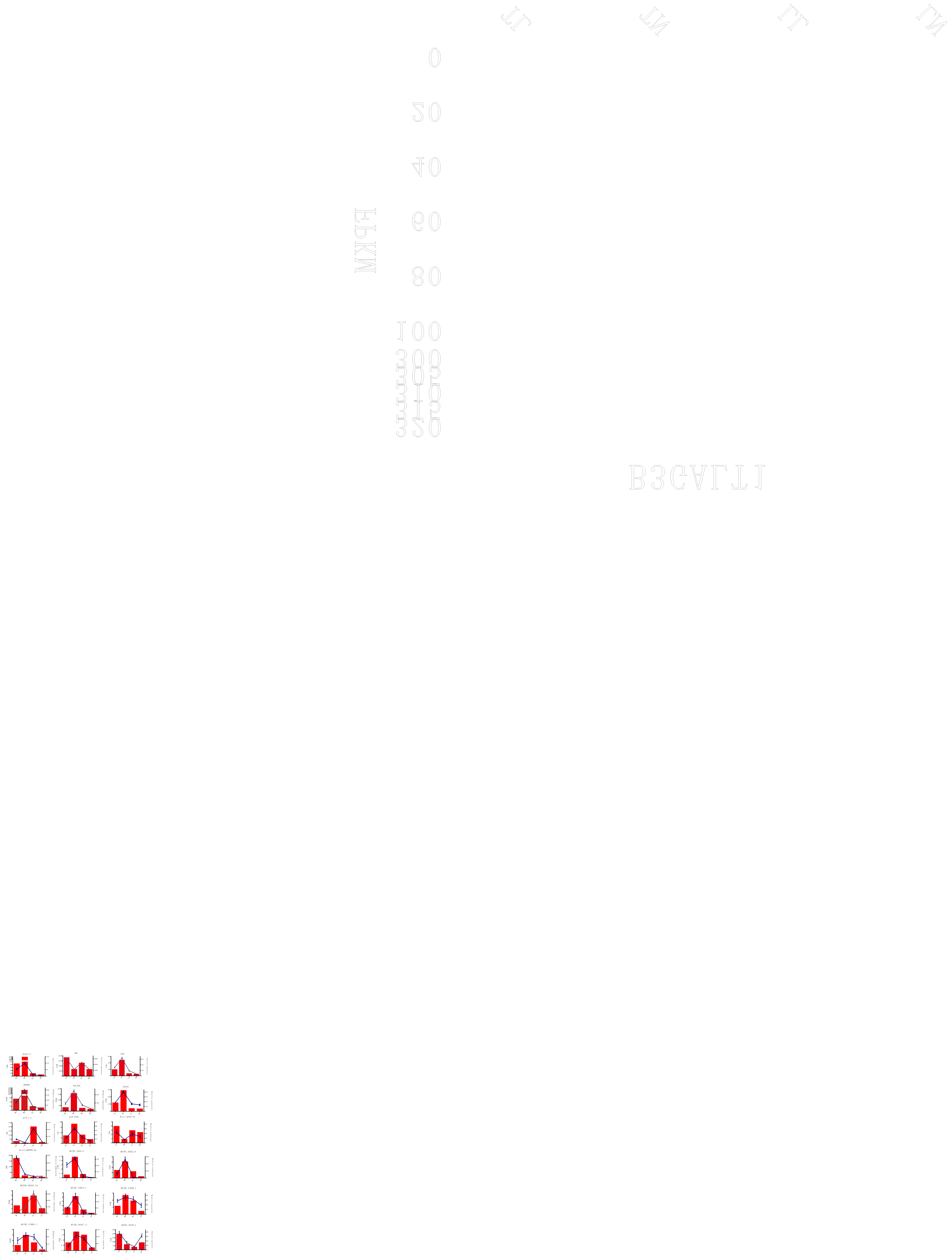


**Figure S2** Expression patterns of randomly selected 6 DEmRNAs, 4 DEmiRNAs, and 8 DElncRNAs. Histogram represent the change in transcript level according to the FPKM value of RNA-seq (left y-axis), and Broken line indicate that relative expression level defense by RT-PCR (right y-axis).


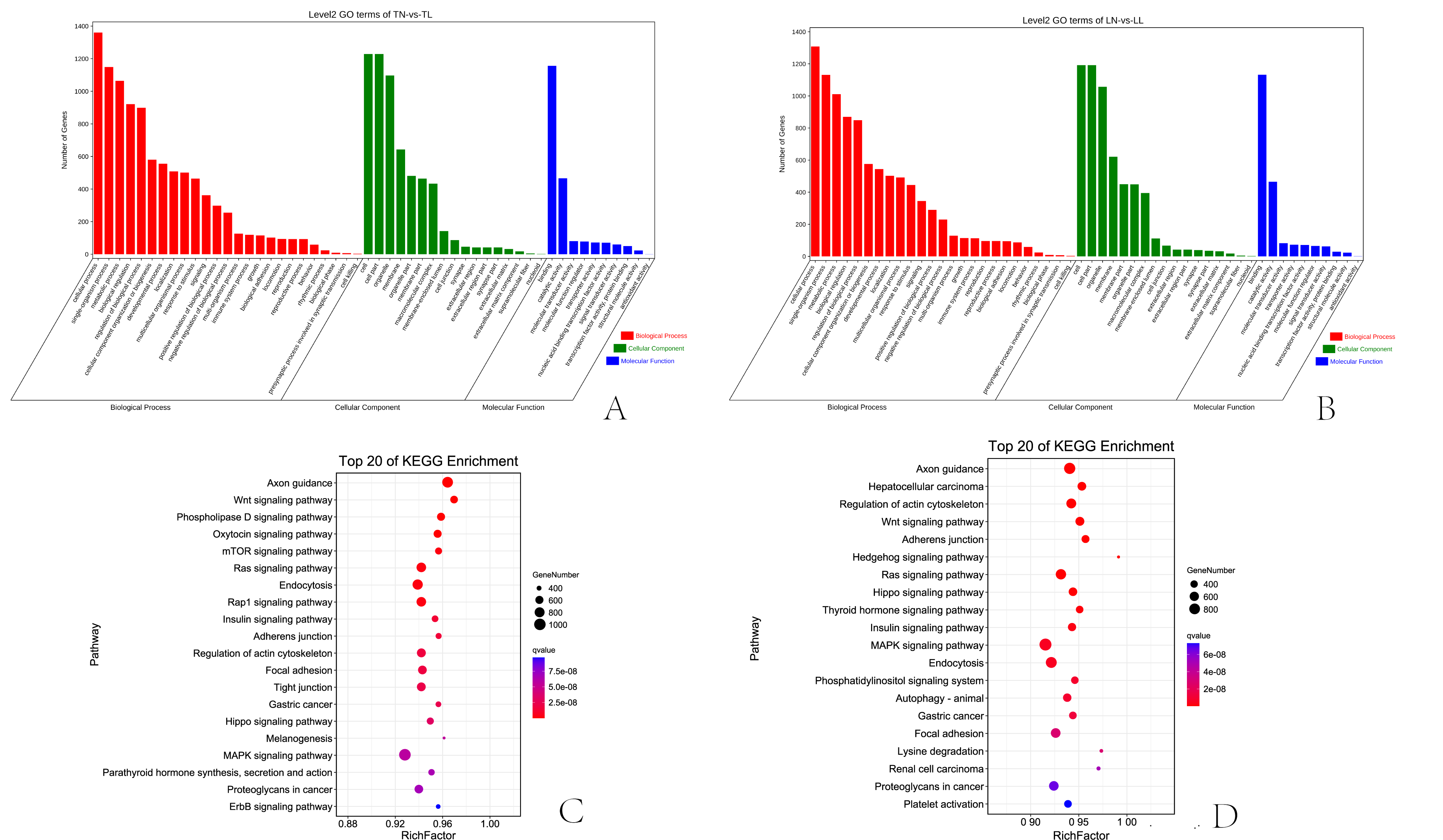


**Figure S3.** Functional annotation analysis of target mRNAs of DEmiRNAs in ATII cells. **A.** Histogram of GO annotation results of target mRNAs of DEmiRNAs between TN and TL groups. **B.** Histogram of GO annotation results of target mRNAs of DEmiRNAs between LN and LL groups. **C.** Top 20 KEGG enrichment pathways of target mRNAs of DEmiRNAs between TN and TL groups. **D.** Top 20 KEGG enrichment pathways of target mRNAs of DEmiRNAs between LN and LL groups.
